# Monitoring Air Quality with Transplanted Bryophytes in a Neotropical Andean City

**DOI:** 10.3390/life11080821

**Published:** 2021-08-12

**Authors:** Ángel Benítez, Lizbeth Armijos, James Calva

**Affiliations:** 1 Biodiversidad de Ecosistemas Tropicales-BIETROP, Herbario HUTPL, Departamento de Ciencias Biológicas y Agropecuarias, Universidad Técnica Particular de Loja, San Cayetano s/n, Loja 1101608, Ecuador; 2 Titulación de Biología, Universidad Técnica Particular de Loja, San Cayetano s/n, Loja 1101608, Ecuador; Lizbeth.arsot@gmail.com; 3 Departamento de Química, Universidad Técnica Particular de Loja, San Cayetano s/n, Loja 1101608, Ecuador; jwcalva@utpl.edu.ec

**Keywords:** active biomonitoring, metals, mosses, *Rhacocarpus purpurascens*, *Sphagnum*, *Thuidium delicatulum*

## Abstract

Air pollution is one of the main global environmental problems, where bryophytes, due to their high capacity to retain metals and other pollutants, have been widely used in active air quality monitoring studies in temperate and tropical zones. Thus, in this study, we analyzed for the first time the concentrations of eight metals (cadmium, copper, nickel, aluminum, iron, manganese, lead and zinc) in three species of transplanted mosses (*Rhacocarpus purpurascens* (Brid.) Paris, *Sphagnum* sp. and *Thuidium delicatulum* (Hedw.) Schimp.) from Ecuador. Significant differences were found for the three species in the concentrations of Al, Mn, Fe and Zn between urban and control areas, pointing to the Central zone as the main source of contamination with the highest concentrations of Al, Fe, Mn and Zn, related to vehicular traffic. Lead did not differ between zones for *Rhacocarpus purpurascens* and *Sphagnum* sp.; however, *Thuidium delicatulum* accumulated different concentrations between urban areas and the control areas. The three species of mosses provided valuable information on the contamination of Al, Fe, Mn, Pb and Zn in the urban area of the city of Loja, and therefore can be used in future air quality monitoring programs over time in tropical cities.

## 1. Introduction

Anthropogenic activities such as industrial discharges, agricultural practices, combustion, vehicular traffic, and poor waste management are the main causes of environmental pollution in urban areas [1,2], causing negative effects on people’s health [3,4,5]. Thus, several countries are researching and implementing strategies aimed at improving environmental quality [6,7].

Vehicular traffic is one of the most important sources of air pollution in urban areas [8] including tropical zones [9]. For instance, previous studies have shown that traffic is a major source of metals [9,10], carbon monoxide (CO) [11], sulfur oxides (SOx), nitrogen oxides (NOx) and particulate matter [12].

In this context, one of the methods to assess air quality is biomonitoring based on the use of biological species to detect air pollutants, allowing the establishment of an environmental quality control program, including air [13]. Biomonitors allow the determination of the location of pollutant sources, distribution patterns and relative deposition intensities [14]. Biomonitoring studies present great advantages in comparison with the use of air filters or air samplers, related to a higher degree of accumulation of metals, and reduce the cost of monitoring and controlling air quality [15,16]. The data collected by active monitoring allow us to solve certain limitations of passive monitoring (native species of an area); for instance, this monitoring can be applied in sites that lack native organisms, allowing the minimization of biological variability by using organisms collected from the same population and facilitating the complete control of the exposure time [17].

Bryophytes take up essential elements directly from the air and have stronger adsorption capacities for metals [18,19,20]; thus, they are suitable biomonitors for detecting air pollutants in urban areas [21,22]. Species of mosses such as *Ceratodon purpureus* (Hedw.) Brid. [23], *Haplocladium microphyllum* (Hedw.) Broth. [24], *Hylocomium splendens* (Hedw.) Schimp. [25,26], *Hypnum cupressiforme* Hedw. [7,27], *Pleurozium schreberi* (Brid.) Mitt. [25], *Scleropodium purum* (Hedw.) Limpr. [28], *Sphagnum denticulatum* Brid. [29], *Sphagnum girgensohnii* Russow. [7,30], *Thuidium delicatulum* (Hedw.) Schimp. [31], and *Thuidium tamariscellum* (Müll. Hal.) Bosch and Sande Lac. [32] have been widely used in active monitoring studies of air pollutants. These studies affirm that urbanized localities are highly affected by the presence of metals (e.g., zinc, cadmium and copper), related to vehicular traffic [8], the coating and automotive industries, as well as the degradation of construction metals and road surfacing materials [26,33,34]. However, most studies have been carried out in temperate zones when compared to tropical zones.

In Ecuador, only one active biomonitoring study has been conducted using mosses as indicators of air pollution in the city of Quito [35], where the authors found the presence of lead and cadmium related to vehicular traffic, but the identification of the species used was not realized. On the other hand, air quality monitoring studies have been carried out in the city of Loja using lichens and bromeliads [36,37]. These studies have shown that urban areas have lower species diversity and a higher accumulation of metals compared to control zones. However, this is the first study to analyze air quality by transplanting bryophytes, which allows the use of low-cost air pollution monitoring systems [15,38]. The present study aims to determine the air quality of the city of Loja by transplanting three species of mosses (*Rhacocarpus purpurascens*, *Sphagnum* sp. and *Thuidium delicatulum*) due to the fact that urban areas of the city of Loja, Ecuador have high levels of air pollution (e.g., metals) related to vehicular traffic [36,37]. We hypothesized that increased urbanization and vehicular traffic towards the center of the city will result in increased bioaccumulation of heavy metals in transplanted mosses.

## 2. Materials and Methods

### 2.1. Study Area

**Figure 1:**
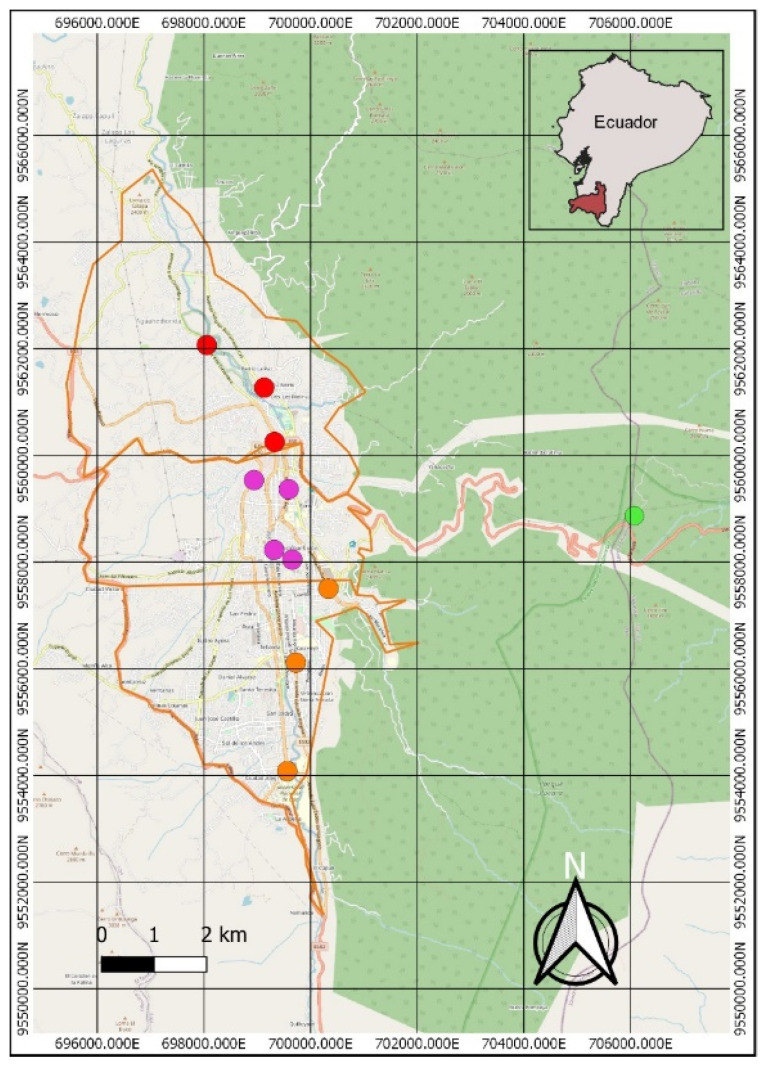
Study area of city of Loja (Southern Ecuador), showing the location of the zones. South zone (orange circle), Central zone (violet circle), North zone (red circle), Control zone (green circle).

The study was carried out in the city of Loja, located in the south of Ecuador at 2100 m a.s.l. For monitoring purposes, the city of Loja was divided into three zones (North, Central and South), with three locations in the North and South zones and four locations in the Central zone (Figure 1). The design has been structured based on previous environmental monitoring studies [37,39,40]. The study was conducted between March and May 2019.

The South zone (S) is characterized by a greater concentration of metals in the air and by recent urban development. Here, Cd, Cu, Mn, Pb and Zn in the air reach values up to 30.83 mg g^−1^, 21.27 mg g^−1^, 53.49 mg g^−1^, 39.48 mg g^−1^, and 91.37 mg g^−1^, respectively. The Central zone (C) is characterized by a high level of air pollution with metals and a high degree of urbanization. In this area, levels of Cd, Cu, Mn, Pb and Zn in the air reach values up to 34.66 mg g^−1^, 25.41 mg g^−1^, 20.03 mg g^−1^, 25.29 mg g^−1^, and 100.54 mg g^−1^, respectively. Finally, the North zone (N) is an urban area with high levels of metals, but the zone still has some recreational parks. In this area, levels of Cd, Cu, Mn, Pb and Zn in the air reach values up to 27.99 mg g^−1^, 31.02 mg g^−1^, 56.81 mg g^−1^, 42.95 mg g^−1^, and 44.46 mg g^−1^, respectively [37].

Transplants were carried out using terrestrial mosses of the species *Rhacocarpus purpurascens*, *Sphagnum* sp. and *Thuidium delicatulum*. Samples were collected from an uncontaminated area in the buffer zone (Control zone: Ctr) of the Podocarpus National Park, which is located on the outskirts of the city (3°59′19″ N, 79°8′38″ E). The moss vouchers were deposited in Herbario de la Universidad Técnica Particular de Loja (HUTPL).

**Figure 2:**
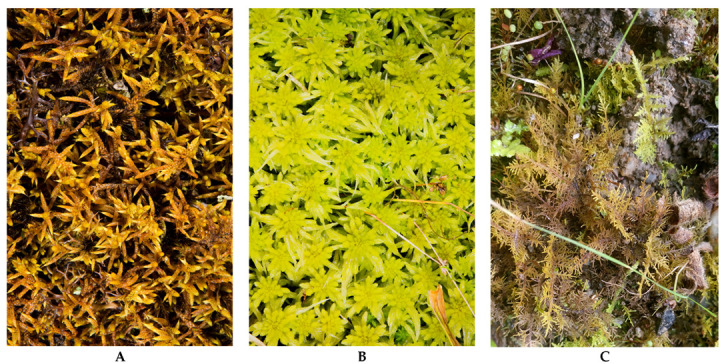
Species used for active monitoring of air quality in city of Loja (Southern Ecuador). (**A**) *Rhacocarpus purpurascens*; (**B**) *Sphagnum* sp.; (**C**) *Thuidium delicatulum*.

*Rhacocarpus purpurascens* (Figure 2A) grows on soil and rocks, and is distributed in the Americas, Africa, Australia and New Zealand [41]. This species is characterized by a unique ultrastructure in the cell walls of its leaves, which makes them highly porous [42]. This species has been used in studies on the essential oil constituents of mosses [43], AB-1300 (HUTPL).

*Sphagnum* sp. (Figure 2B) is the most abundant genus of mosses and is widely distributed throughout the world, forming cushions on rocks, trunks and soils. *Sphagnum* sp. has been widely used as a bioindicator of air pollution [8,44,45,46] due to the high number of pores in the hyalocysts [47], which help to accumulate metals related to air pollution, AB-1301 (HUTPL).

**Figure 3:**
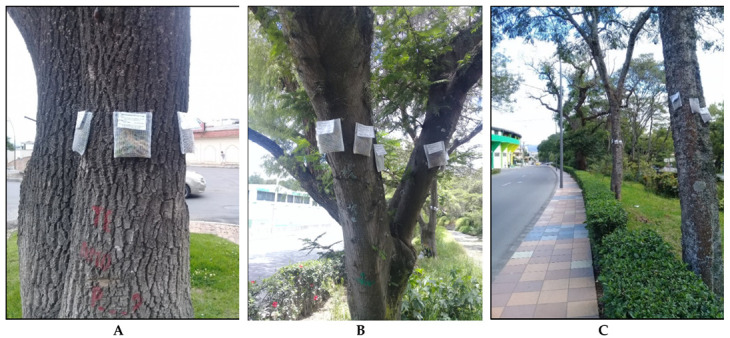
Transplanting of samples of the three species used for active monitoring in the city of Loja. (**A**) South zone, (**B**) Central zone, and (**C**) North zone.

*Thuidium delicatulum* (Figure 3C) grows on rocks and trunks, in shady and dry zones, and it has an extensive branching that allows a large exposed area for ion exchange [48]. *T*. *delicatulum* has been used as a bioindicator of metal deposition [31,49], AB-1302 (HUTPL).

### 2.2. Design and Data Collection

The collected terrestrial mosses were transported to the laboratory in order to manually remove soil particles and plant debris, then left to dry in the open air, and the material of each species was mixed separately by hand in order to obtain homogenized samples of each species. The bag material, the mesh size and the amount of plant material were selected according to the protocol of Ares et al. [38]. We took 0.5 g of moss, which was placed in 10 × 12 cm nylon net bags with a mesh size of 2 mm. A total of 15 moss bags for each species were controls (Ctr), which were treated in the same way as the transplants but were not exposed to air pollution. These samples were stored at room temperature under laboratory conditions as a control sample for the determination of initial contaminant concentrations [29]. At each locality, 15 bags (5 bags of each species) were attached perpendicular to a tree trunk, at a height of 2–3 m [38], thus obtaining a total of 180 bags throughout the city (Figure 3). The exposure of the bags lasted for 90 days (March to May 2019), after which time they were removed for further analysis in the laboratory.

We obtained traffic flow with punctual one-day sampling data related to the number of vehicles for each zone, following the same protocol of Käffer et al. [50] and Hu et al. [8] with few adaptations. Three different categories were considered: (LV) = light vehicles (cars and small vans), (HV) = heavy vehicles (trucks and buses) and (MT) = motorbikes.

### 2.3. Elemental Bioccumulation

For the chemical analysis, the samples were sieved to remove the residues, and then the samples were dried in a drying oven at 50 °C. The microwave digestion system MARS (Microwave Accelerated Reaction System) 6 by CEM Corporation was used [51]. The digestion method requires the addition of 0.5 g of sample and 10 mL of HNO3 in the digestion vessel [52]. After the digestion, the volume of each sample was adjusted to 100 mL using double deionized water. The content of cadmium (Cd), copper (Cu), nickel (Ni), aluminum (Al), iron (Fe), manganese (Mn), lead (Pb) and zinc (Zn) in the samples was analyzed using atomic absorption spectroscopy (AAnalyst 400; Perkin Elmer Sdn Bhd, Selangor, Malaysia). Calibration curves were prepared with certified standards (Merck KGaA, Darmstadt, Germany) for each of the metals analyzed.

### 2.4. Data Analysis

To evaluate the changes in the concentration of metals in each of the zones, parametric and nonparametric statistical tests were performed based on the Shapiro–Wilk normality test. Cadmium (Cd), copper (Cu) and nickel (Ni) showed values of zero for the control samples and the samples transplanted in the city; thus, these metals were not considered in the statistical analyses. For the three species, a one-way analysis of variance was performed (ANOVA) for metals that met normality assumptions (Shapiro–Wilk, *p*-value > 0.05) and the nonparametric Kruskal–Wallis test for those that did not have a normal distribution (Shapiro–Wilk, *p*-value < 0.05). In addition, to identify significant differences in metal accumulation between zones, the Tukey HSD post hoc multiple comparison test was implemented as a parametric test, and Dunn’s non-parametric test with the Dunn test package was used [53]. In order to identify correlation between vehicular traffic and metal content in three moss species, Pearson correlation (normal distribution) and Spearman correlation (no-normal distribution) analyses were applied. All analyses were performed using the statistical software Rstudio version 1.1.453 [54].

## 3. Results

The mean concentration of most of the metals was higher in the urbanized areas compared to the control samples for the three species (Table 1).

**Figure 4:**
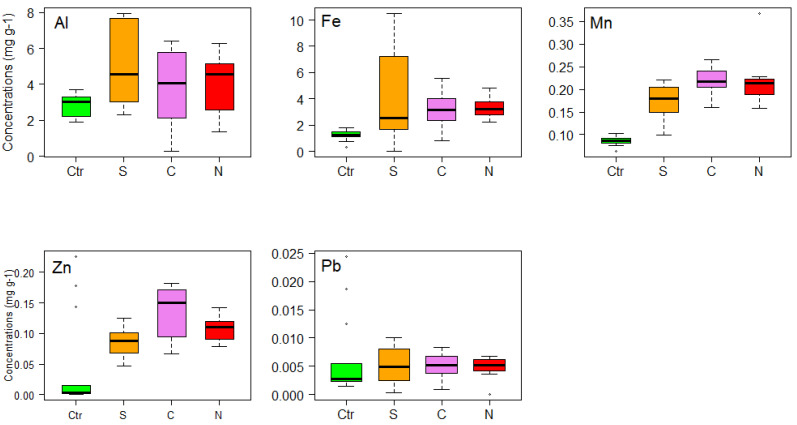
Box plots of the concentrations (mg g−^1^) of metals (Al, Fe, Mn, Pb and Zn) in *Rhacocarpus purpurascens*, used for active monitoring of air quality in city of Loja (Southern Ecuador). Ctr = Control, S = South, C = Central, N = North.

For *Rhacocarpus purpurascens*, values of Al, Fe, Mn and Zn were high for urban areas in comparison with the control samples (Figure 4). On the other hand, Al and Fe presented high values in the South zone, followed by the North and Central zones, but for zinc, high values were registered in the Central zone. *R*. *purpurascens* showed that there was a significant positive correlation between Fe, Mn and Zn concentrations associated with vehicular traffic (Table 1).

**Figure 5:**
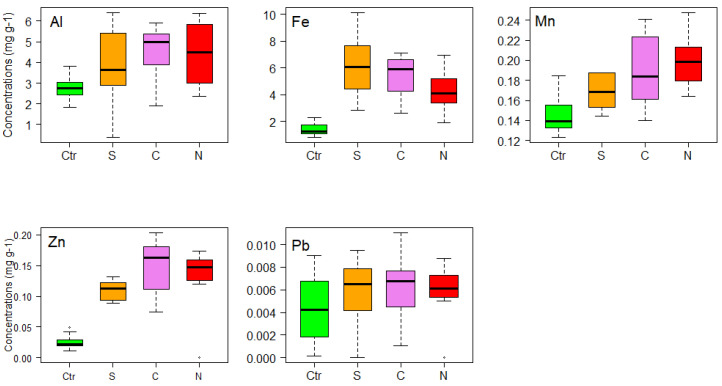
Box plots of the concentrations (mg g−^1^) of metals (Al, Fe, Mn, Pb and Zn) in *Sphagnum* sp., used for active monitoring of air quality in city of Loja (Southern Ecuador). Ctr = Control, S = South, C = Central, N = North.

Following the same pattern, for *Sphagnum* sp. Al, Fe, Mn and Zn had the highest values in the urban areas compared to the control samples (Figure 5). Aluminum, manganese and zinc presented high values in the Central zone, followed by North and South zones; however, for iron, high values were registered in the South zone of the city, followed by Central and North zones. The results for *Sphagnum* sp. showed that there was a significant positive correlation between Al, Fe, Mn and Zn concentrations associated with vehicular traffic (Table 1).

**Figure 6:**
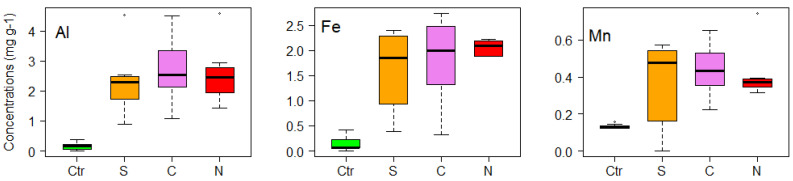

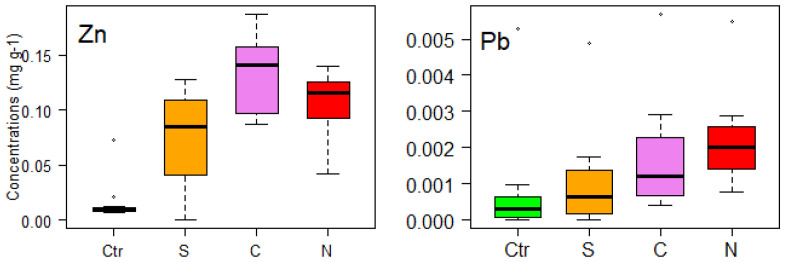
Box plot representation of the concentrations (mg g^−1^) of metals (Al, Fe, Mn, Pb and Zn) in *Thuidium delicatulum*, used for active monitoring of air quality in city of Loja (Southern Ecuador). Ctr = Control, S = South, C = Central, N = North.

In *Thuidium delicatulum*, aluminum, manganese and zinc presented high values in the Central zone, followed by the North and South zones; however, for iron, the highest values were recorded in the North zone of the city, followed by the Central and South zones (Figure 6). The results for *T*. *delicatulum* showed that there was a significant positive correlation between Al, Fe, Mn, Pb and Zn concentrations and vehicular traffic (Table 1).

For the three moss species, there were significant differences in the concentrations of aluminum (Al), manganese (Mn), iron (Fe) and zinc (Zn) between the urbanized areas and the control samples. Lead (Pb) concentrations did not show significant differences for the control samples for *R*. *purpurascens* and *Sphagnum* sp., while for *T*. *delicatulum* the differences were significant (Table 1). On the other hand, the Tukey HSD test and Dunn test performed on the species *R*. *purpurascens* showed significant differences between the accumulation of metals in the control samples and urban areas studied (South, Central, North) for Fe and Mn (Table 2).

In the species *Sphagnum* sp., the Tukey HSD test and the Dunn test showed significant differences between the accumulation of metals in the control samples and the urban zones (South, Central, North) for Al, Fe, Mn and Zn (Table 3).

In the species *T*. *delicatulum*, the Tukey HSD and Dunn test showed significant differences between the accumulation of Al, Fe, Mn, Pb, and Zn in the control samples and the urban areas (Table 4).

## 4. Discussion

For the three moss species, there were significant differences in the concentrations of aluminum (Al), manganese (Mn), iron (Fe) and zinc (Zn) between the urban areas (South, Central and North) and the control samples. Similar to our results, previous studies have shown that the highest concentrations of these elements were observed in areas characterized by the intense flow of public transportation [4,8,31,47,55]. For instance, Capozzi et al. [10] and Hu et al. [8] showed that a high metal concentration (e.g., Zn) in *Hypnum cupressiforme* and *Sphagnum junghuhnianum* is related to vehicular traffic.

In addition, corroborating this pattern, passive monitoring studies using lichens and bromeliads in the city of Loja have identified urban areas with high levels of contaminants such as zinc and manganese [36,37]. All three moss species point to the center area as a focus of contamination for the metals aluminum, manganese, and zinc; thus, an increase in these metals (Al, Mn, and Zn) is related to road dust resuspension, vehicle brake abrasion, and tire wear [56,57,58,59,60,61]. On the other hand, the concentrations of lead (Pb) did not show significant differences between control and urban areas for *Rhacocarpus purpurascens* and *Sphagnum* sp., due to the fact that industrial areas report higher Pb concentration levels than areas with high vehicular traffic [62]—as in our case, the city of Loja has a low level of industrial development.

For *Rhacocarpus purpurascens*, significant differences were shown between the accumulation of metals in the control samples and urban areas (South, Central, North) only for two of the metals analyzed (Fe, Mn). This suggests that the species has a low capacity to retain certain metals; this may be due to the structure of the cell walls of the leaves, which are highly porous and thus water can easily penetrate the reticular layer, increasing the likelihood that certain metals are leached [24,63]. In *Sphagnum* sp., significant differences were found for Al, Fe, Mn and Zn between the control samples and the urban areas (South, Central, North). *Sphagnum* sp. has a high capacity to retain metals related to vehicular traffic, as shown by previous studies that have observed positive results in terms of the accumulation of Al, Fe, Mn, Pb and Zn in urban areas [8,18,45,61,64,65]. However, Pb concentration in this study was not significant for two species. This result can be attributed to external factors such as climatic conditions, the mineral composition of soil dust, the natural element cycling process and the vegetation zone, which have a significant influence on the efficiency of metal uptake in mosses [66,67,68].

On the other hand, for *Thuidium delicatulum* Al, Fe, Mn, Pb and Zn showed significant differences between control samples and the urban areas. These results are in agreement with Rodríguez-Quiel et al. [31] and Castello et al. [69], who showed that the method of transplanting samples of *T*. *delicatulum* and *Pseudoscleropodium purumis* is effective for determining the variation of Al, Fe, Pb and Zn as the main air pollutants. Likewise, our results show that of the three mosses used in the study, *T*. *delicatulum* showed significant differences for lead, which indicates that the physiology and morphology of mosses are involved in the process of bioconcentration and absorption of pollutants [48,70].

## 5. Conclusions

The three urban zones of the city of Loja showed higher concentrations of Al, Fe, Mn and Zn compared to the control samples for the three species of mosses (*Rhacocarpus purpurascens*, *Sphagnum* sp., and *Thuidium delicatulum*) related to vehicular traffic. *Sphagnum* sp. and *T*. *delicatulum* point to the Central zone as the main source of contamination, with the highest concentrations of Al, Fe, Mn and Zn. Thus, this study might serve as a reference for future investigations on the bioaccumulation of pollutants in *Rhacocarpus purpurascens*, *Sphagnum* sp., and *Thuidium delicatulum* in similar urban tropical areas.

## Figures and Tables

**Table 1:** Mean concentration, standard deviation and *p*-value (ANOVA and Kruskal–Wallis) of Al, Fe, Mn, Pb and Zn in *Rhacocarpus purpurascens*, *Sphagnum* sp. and *Thuidium delicatulum* in the city of Loja (mg g^−1^). r = correlation coefficient between vehicular traffic and metals for each species.

Species	Metal	Control	South	Central	North	*p* Value	r	*p* Value
*Rhacocarpus purpurascens*	Al	2.783 ± 0.623	5.090 ± 2.339	3.876 ± 2.066	3.948 ± 1.902	0.041	0.246	0.115
	Fe	1.228 ± 0.372	4.170 ± 3.674	3.064 ± 1.366	3.331 ± 0.867	0.0004	0.398	0.009
	Mn	0.086 ± 0.010	0.173 ± 0.040	0.221 ± 0.030	0.223 ± 0.069	<0.0001	0.801	<0.0001
	Pb	0.006 ± 0.007	0.005 ± 0.003	0.005 ± 0.002	0.005 ± 0.002	0.696	−0.160	0.309
	Zn	0.042 ± 0.078	0.086 ± 0.026	0.131 ± 0.044	0.107 ± 0.023	0.0050	0.5652	<0.0001
*Sphagnum* sp.	Al	2.771 ±0.570	3.718 ± 2.109	4.545 ± 1.335	4.429 ± 1.5342	0.0124	0.485	0.002
	Fe	1.368 ± 0.411	6.197 ± 2.553	5.396 ± 1.596	4.281 ± 1.5420	<0.0001	0.789	<0.0001
	Mn	0.147 ± 0.023	0.186 ± 0.057	0.241 ± 0.168	0.188 ± 0.0527	0.012	0.343	0.041
	Pb	0.004 ± 0.003	0.006 ± 0.003	0.006 ± 0.003	0.006 ± 0.0026	0.44	0.278	0.099
	Zn	0.026 ± 0.010	0.110 ± 0.017	0.149 ± 0.047	0.130 ± 0.055	<0.0001	0.831	<0.0001
*Thuidium delicatulum*	Al	0.151 ± 0.107	2.312 ± 1.048	2.750 ± 1.061	2.560 ± 1.027	<0.0001	0.803	<0.0001
	Fe	0.309 ± 0.646	3.453 ± 5.558	2.238 ± 1.701	2.176 ± 1.11	<0.0001	0.368	0.022
	Mn	0.132 ± 0.011	0.367 ± 0.239	0.441 ± 0.127	0.414 ± 0.148	<0.0001	0.687	<0.0001
	Pb	0.001 ± 0.002	0.001 ± 0.002	0.002 ± 0.002	0.002 ± 0.002	0.0035	0.271	0.039
	Zn	0.015 ± 0.018	0.075 ± 0.047	0.131 ± 0.035	0.105 ± 0.033	<0.0001	0.768	<0.0001

**Table 2:** Post hoc Tukey’s test and Dunn’s test for metal accumulation in *R*. *purpurascens* according to the different study areas; Est = statistic; *p* < 0.05 is considered significant; Ctr = Control, S = South, C = Central, N = North.

Tukey Test	Al		Dunn Test	Fe		Mn		Pb		Zn	
Zone	Est	*p*-Value	Zone	Est	*p*-Value	Est	*p*-Value	Est	*p*-Value	Est	*p*-Value
N-C	0.073	1.000	N-C	−0.567	1	0.445	1	0.107	1	0.768	1
S-C	1.214	0.422	S-C	0.010	1	1.741	0.2452	0.248	1	1.537	0.371
Ctr-C	−1.092	0.379	T-C	3.391	0.0021	5.137	< 0.0001	1.113	0.797	3.490	0.0015
S-N	1.142	0.593	S-N	0.523	1	1.108	0.8034	0.1181	1	0.639	1
Ctr-N	−1.165	0.484	T-N	3.396	0.0021	3.823	0.0004	0.8176	1	2.126	0.101
Ctr-S	−2.307	0.025	T-S	2.936	0.0100	2.700	0.0208	0.7160	1	1.413	0.421

**Table 3:** Post hoc Tukey’s test and Dunn’s test for metal accumulation in *Sphagnum* sp. according to the different study zones; Est = statistic; *p* < 0.05 is considered significant; Ctr = Control, S = South, C = Central, N = North.

Tukey Test	Al		Fe		Pb		Dunn Test	Mn		Zn	
Zone	Est	*p*-Value	Est	*p*-Value	Est	*p*-Value	Zone	Est	*p*-Value	Est	*p*-Value
N-C	−0.117	1	−1.115	0.9650	−4.413	0.990	N-C	−0.273	1	0.807	1
S-C	−0.827	0.654	0.8008	0.3849	−4.689	0.990	S-C	0.483	1	1.348	0.5335
Ctr-C	−1.774	0.023	−4.028	<0.0001	−1.917	0.462	T-C	2.539	0.033	4.284	0.0001
S-N	−0.711	0.751	1.9125	0.1913	−2.767	0.999	S-N	0.736	1	0.601	1
Ctr-N	−1.657	0.034	−2.912	<0.0001	−1.475	0.670	T-N	2.847	0.013	3.373	0.0022
Ctr-S	−0.947	0.464	−4.829	0.0130	−1.448	0.742	T-S	1.772	0.229	2.399	0.049

**Table 4:** Post hoc Tukey’s test and Dunn’s test for metal accumulation in *T*. *delicatulum* according to the different study zones; Est = statistic; *p* < 0.05 is considered significant; Ctr = Control, S = South, C = Central, N = North.

Dunn Test	Al		Fe		Mn		Pb		Tukey Test	Zn	
Zone	Est	*p*-Value	Est	*p*-Value	Est	*p*-Value	Est	*p*-Value	Zone	Est	*p*-Value
N-C	0.251	1	−0.183	1	0.554	1	−0.715	1	N-C	−0.026	0.395
S-C	0.801	1	0.238	1	0.735	1	1.603	0.3441	S-C	−0.056	0.005
Ctr-C	4.449	<0.0001	3.512	0.0013	3.890	0.0003	2.847	0.0145	T-C	−0.115	<0.0001
S-N	0.484	1	0.385	1	0.143	1	2.107	0.1136	S-N	−0.030	0.317
Ctr-N	3.649	0.0005	3.273	0.0032	2.852	0.0130	3.225	0.0043	T-N	−0.089	<0.0001
Ctr-S	3.253	0.0034	2.974	0.0088	2.809	0.0149	0.972	0.9956	T-S	−0.059	0.002

## Data Availability

Data is contained within the article.
